# Systematic Review of Patient-Reported Outcome Measures in Locally Recurrent Rectal Cancer

**DOI:** 10.1245/s10434-023-13388-5

**Published:** 2023-04-18

**Authors:** Niamh McKigney, Fergus Houston, Ellen Ross, Galina Velikova, Julia Brown, Deena Pravin Harji

**Affiliations:** 1 Clinical Trials Research Unit, Leeds Institute of Clinical Trials Research, University of Leeds, Leeds, UK; 2 Princess Alexandra Hospital, Brisbane, Australia; 3 Inverclyde Royal Hospital, Greenock, Scotland, UK; 4 Leeds Institute of Medical Research at St James’s, University of Leeds, Leeds, UK; 5 St. James’s Institute of Oncology, St. James’s University Hospital, Leeds, UK; 6 Department of Colorectal Surgery, Manchester University NHS Foundation Trust, Manchester, UK

## Abstract

**Background:**

The availability of high-quality patient-reported outcome (PRO) data is crucial to guiding shared decision-making in the context of locally recurrent rectal cancer (LRRC), where potential treatment benefits must be balanced against the impact of both the disease and treatment on PROs, such as quality of life. This review aimed to identify the patient-reported outcome measures (PROMs) currently being reported in LRRC and to appraise the methodological quality of studies using these measures.

**Methods:**

PubMed, Embase and CINAHL databases were searched, including studies published up until 14^th^ September 2022. Studies in adults with LRRC reporting PROMS as a primary or secondary outcome measure were included. Data were extracted concerning the methodological quality of the reporting of PROMs using criteria informed by the CONSORT-PRO checklist and the psychometric properties of the PROMs identified using the COSMIN Risk of Bias checklist.

**Results:**

Thirty-five studies including 1914 patients with LRRC were identified. None of the studies included in the review met all eleven criteria for the quality of reporting of PROMs. Seventeen PROMs and two clinician-reported outcome measures were identified, none of which have been validated for use in patients with LRRC.

**Conclusions:**

None of the PROMs which are currently being used to report PROs in LRRC have been validated for use in this cohort of patients. Future studies in this disease area should focus on utilising PROMs that have undergone a robust development process including patients with LRRC, to produce data which is high quality, accurate and relevant.

**Supplementary Information:**

The online version contains supplementary material available at 10.1245/s10434-023-13388-5.

The availability of high-quality studies reporting patient-reported outcome (PRO) data utilising robustly developed patient-reported outcome measures (PROMs), offer several advantages to patient care, including their utility within shared decision-making discussions. Baseline PRO data has been shown to act as a prognostic factor for overall survival in cancer patients,^1^ including those with advanced malignancy.^2,3^ Integrating PROs into clinical care to monitor adverse effects of cancer treatment can also enhance patient quality of life,^4^ and has even been reported to improve survival.^5,6^ The interest in utilising PROMs from both a clinical and academic standpoint continues to grow given the potential utility of these outcome measures, including in patients with locally recurrent rectal cancer (LRRC). The inclusion of patient-reported outcomes (PROs) is particularly important in the context of advanced malignancy such as LRRC. LRRC can lead to debilitating symptoms such as pain, bleeding/discharge from the rectum, pelvic sepsis, urinary symptoms, lower limb symptoms and impaired sexual function. Surgical resection represents the only curative treatment option for patients with LRRC, with 5-year survival rates of 42.4% - 63% reported by specialist tertiary centres.^7–11^ Exenterative surgery has evolved, with ultra-radical techniques developed in recent years, which can offer potential cure to patients with LRRC, such as high sacrectomy and extended lateral pelvic sidewall excision (ELSiE), are generally accompanied by significant morbidity.^12–14^ In this context, balancing the patients’ existing symptoms, the potential survival benefits to be gained from treatment and their impact on PROs, is essential to enabling patients to make informed decisions regarding their care.

However, it is crucial that the methodological quality of the studies reporting PROs and the PROMs used are sufficient to produce valid and reliable results, particularly in complex disease settings. Validity is the degree to which a PROM measures the construct it purports to measure.^15^ In a clinical context, such as in measuring health-related quality of life (HrQoL) in patients with LRRC, a PROM can only be considered valid if there is evidence that it has been developed with input from patients with LRRC and provides a comprehensive assessment of HrQoL as the construct of interest, meaning that all aspects of HrQoL that are relevant to patients with LRRC are included. PROMs can be designed as disease-specific or generic, for instance, a generic PROM measures concepts which are broadly relevant to the population, whereas disease-specific PROMs measure concepts specific to a group of patients with a particular condition. To be considered valid in a specific group of patients, both disease-specific and generic PROMs should be shown to have content validity in the group of patients they have been designed for.

The existing evidence concerning PROs in LRRC possesses several limitations from a methodological standpoint, this includes heterogeneity in relation to the groups of patients included, with outcomes frequently reported in combined cohorts of patients with primary and recurrent disease,^16–19^ and heterogeneity in comparator groups. In addition to significant variability in the PROMs used and timing of PROM assessment.^16–19^ The majority of existing studies are retrospective in nature^18^ and the evidence is generally low in quality.^16–20^ Denys et al.’s review focused on patient-centred outcomes following pelvic exenteration for colorectal cancer, including both primary and recurrent disease, also found that the impact of urinary complications, discomfort or pain on sitting and functional disability are inadequately represented in the PROMs currently being used.^19^

This review sought to evaluate the methodological quality of the existing evidence concerning PROs in LRRC, utilising a systematic approach. The specific aims of the review were to identify the PROMs currently being used to report outcomes in patients with LRRC and to examine the methodological quality of the studies against criteria informed by the Consolidated Standards of Reporting Trials- Patient Reported Outcome (CONSORT-PRO) extension,^21,22^ and the psychometric properties of the PROMs identified using the COnsensus-based Standards for the selection of health Measurement INstruments (COSMIN) Risk of Bias checklist.^23,24^

## Methods

This systematic review was conducted using a pre-specified protocol in keeping with Cochrane guidelines,^25^ and reported in line with the Preferred Reporting Items for Systematic reviews and Meta-Analyses (PRISMA) checklist.^26^ The review was registered on the international prospective register of systematic reviews, PROSPERO (reference: CRD42022332577).

### Eligibility Criteria

Studies in adults (aged ≥ 18) with LRRC that included PROMs as a primary or secondary outcome measure were included. Studies in patients with LRRC undergoing any form of treatment with curative or palliative intent, were eligible for inclusion. Studies in patients with a history of only local excision for primary rectal cancer who developed a regrowth or recurrence were excluded. Only studies published in the English language were considered. Case reports, conference abstracts, study protocols, reviews and letters were excluded.

### Information Sources

The search was undertaken using the PubMed, Embase and CINAHL databases, including studies published from 1966 (PubMed), 1980 (Embase) and 1981 (CINAHL) up until 14^th^ September 2022. The search strategy can be found in the supplementary material. Reference searching was also undertaken to identify additional studies. Studies describing the psychometric properties of the PROMs identified from this search were retrieved from citations and through manual searching to enable evaluation of the psychometric properties of the PROMs identified.

### Selection Process

Titles and abstracts of studies retrieved were exported to EndNote X9 (Clarivate Analytics, Philadelphia, USA) and duplicates removed. The titles and abstracts were uploaded to Rayyan online software and screened for relevance by two authors (NM and ER). The full text for potentially eligible studies were retrieved and assessed, any queries regarding the eligibility of a study were resolved through discussion with senior authors.

### Data collection process

Data concerning the characteristics of the studies included and the quality of the reporting of PROMs against criteria informed by the CONSORT-PRO checklist were extracted independently by authors NM and ER into Excel®. The COSMIN Risk of Bias checklist^23^ was completed using the Excel® template available from the COSMIN website^27^ independently by authors NM and FH. Any differences in data extraction or ratings were discussed with senior authors to reach consensus.

### Data Items

#### Quality of Reporting of PROMs

There are currently no checklists available via the Enhancing the QUAlity and Transparency Of health Research (EQUATOR) network regarding the inclusion of PRO data for observational studies. The CONSORT-PRO extension was developed to promote transparent reporting of trials including PROs as primary or secondary outcomes; facilitating the interpretation or PRO results for use in clinical practice.^22^ The CONSORT-PRO checklist was used to inform the evaluation of studies identified in relation to how the findings were reported and whether the methodology of the study and the PROMs used were sufficient to capture significant and meaningful findings.

#### PROM Psychometric Properties

**Fig. 1:**
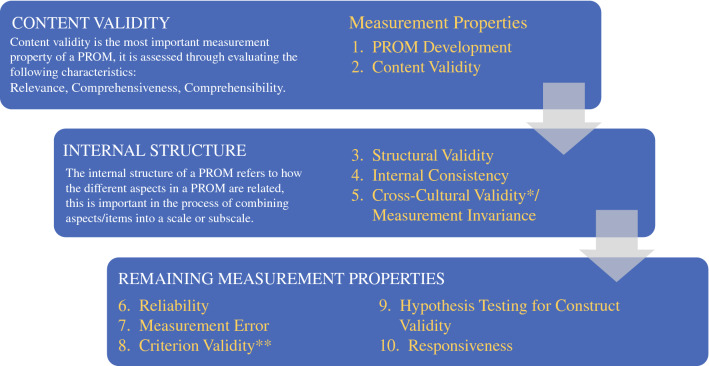
Summary of the COSMIN Risk of Bias Checklist. *Cross-cultural validity was not assessed in this review as the search strategy was not deemed suitable for identifying all studies describing this psychometric property. **The COSMIN panel determined that no gold standard exists for PROMs^30^ and therefore criterion validity was not assessed in this review.

The psychometric properties of the PROMs identified were evaluated using the COSMIN Risk of Bias checklist. The COSMIN Risk of Bias checklist for systematic reviews was developed to assess risk of bias of studies on measurement properties of PROMs,^23^ this information can be used to identify the most appropriate PROM for a specific purpose or study. There are ten criteria (see Figure 1), PROM development and content validity are the first to be assessed, if a PROM is deemed to have insufficient content validity, it should not undergo further assessment. Once sufficient evidence for content validity has been identified, the internal structure and remaining measurement properties are assessed. Studies are qualitatively summarised to give an overall rating of sufficient (+), insufficient (-), inconsistent (±), or indeterminate (?) for each measurement property.^28^ The quality of the evidence is rated using a modified Grading of Recommendations Assessment, Development and Evaluation (GRADE) approach.^29^

### Risk of Bias Assessment

Risk of bias was assessed using the Risk Of Bias In Non-randomized Studies of Interventions (ROBINS-I) tool,^31^ and the revised tool to assess Risk of Bias in randomised trials (RoB 2).^32^

### Data Synthesis

A basic descriptive analysis was undertaken to report the number of patients included in the studies identified and the proportion of patients with LRRC and who contributed to assessments with PROMs.

## Results

### Study Selection

**Fig. 2:**
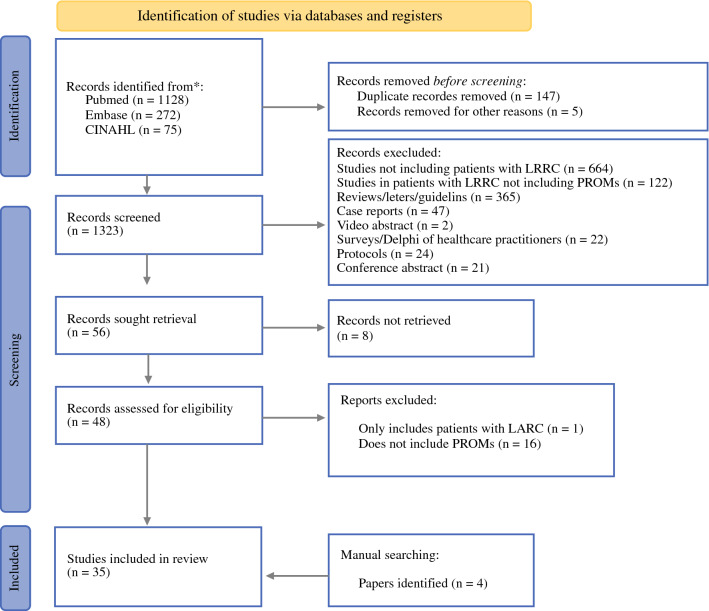
PRISMA flow diagram

A total of 1475 references were identified; 147 duplicates and 5 animal studies were removed. Abstracts were screened for 1323 references and the full text for 56 references were retrieved. Thirty-one eligible references were included from the search strategy in addition to 4 references identified through manual searching (see Figure 2).

### Study Characteristics

A summary of the characteristics of the studies is presented in Table 1, including a total of 1914 patients with LRRC across the 35 studies included, of which PROM data was reported for 1104 (57.7%) patients. Twenty-one (63.6%) of the studies identified were published in the last decade. The studies were conducted mostly in Europe (*n *= 18, 51.4%), Australia (*n* = 13, 37.1%) or the USA (*n* = 4, 11.4%), with one study conducted in China (2.9%). Twenty-six (74.3%) studies recruited patients from a single centre. The majority were prospective cohort studies (*n *= 19, 54.3%) in addition to cross-sectional (*n *= 7, 20.0%), case-control (*n *= 5, 14.3%), retrospective cohort (*n *= 2, 5.7%) and randomised studies (*n *= 2, 5.7%). Eight (22.9%) of the studies identified included only patients with LRRC, in addition to two (5.7%) case control studies comparing patients with LRRC to other cohorts, with sample sizes of patients with LRRC ranging from 12 to 117 patients. The other 23 (69.7%) studies included combined cohorts of patients with primary and recurrent pelvic disease including LRRC, with sample sizes ranging from 12 to 710 patients in total. Median number of PROM assessments was two (IQR 1). In the 19 prospective, longitudinal studies identified, median follow-up was 12 months (IQR 15) the longest follow-up time point was 8 years.^33^

**Table 1:** Summary of studies identified

	Country	Type of study	Primary outcome(s)	Total no patients	Patients included	Total no with LRRC	Total no with LRRC with PRO data	PROM data for LRRC	Inclusion of comparative group	Timing of PROM assessment	PROMs used
Huang 2022 ^7^	Australia	Prospective cohort	QoL	271	PE 2008-2019	160	150	Yes	LARC vs LRRC	Baseline, 6, 12 months	FACT-C SF-36
Westerduin 2021 ^34^	Netherlands, Belgium, and France	Cross-sectional	QoL	52	Redo anastomosis 2007-2017	2	2	No	Control group of 118 patients undergoing TME surgery for rectal cancer	Cross-sectional	LARS EORTC-C30 EORTC-CR29
Alahmadi 2021 ^35^	Australia	Prospective cohort	QoL, Survival, Post-operative complications	710	PE 1994-2019	235	Not known	No	Elderly (>65) vs younger patients undergoing PE	Baseline, 6, 12, 18, 24, 30, 36, 48, 60 months	FACT-C SF-36
McCarthy 2020 ^36^	Australia	Cross-sectional	QoL, lower limb motor, bowel, bladder, and sexual function	256	PE with sacrectomy 2008-2015	111	11	No	PE and sacrectomy vs PE only	Cross-sectional	SF-36 EORTC-C30 & CR29 MSTS LEFS SHIM FSFI
Van Ramshort 2020 ^37^	Australia	Prospective cohort	Flap-related complications	87	PE with VRAM reconstruction 2003-2016	30	Not known	No	PE with VRAM vs PE no VRAM	Baseline, 6, 12, 18, 24 months	FACT-C SF-36
Denost 2020 ^38^	France Australia	Prospective cohort	Surgical resection rate	154	LARC or LRRC 2015-2017	105	Not known	No	PE vs no PE	6, 12 months	SF-36 Distress thermometer Scale
Smith 2020 ^39^	UK	Prospective cohort	Local control	30	SBRT for LRRC 2015-2019	30	30	Yes	No	Baseline 1, 3, 6 months, then 6 monthly intervals	EQ-5D EQ-VAS
Brown 2019 ^40^	Australia	Prospective cohort	Survival, function, QoL	68	Sciatic and femoral nerve resection 1994-2018	33	Not known	No	Complete vs partial sciatic or femoral nerve resection	Baseline, 6, 12 months	FACT-C SF-36
Steffens 2018 ^41^	Australia	Prospective cohort	Survival, QoL	515	PE 1994-2016 (PE 2008-2016 for QoL study)	181	119	No	No	Baseline, 6, 12, 18, 24, 30, 36, 48, 60 months	FACT-C SF-36
Lim 2018 ^42^	Australia	Prospective cohort	Post-operative pain, pre-operative opiate use, post-operative pain	99	PE 2013-2014	51	42	Yes	No	Days 1, 2, 3 and 7	VNRS
Choy 2017 ^43^	Australia	Prospective cohort	QoL	117	LRRC referred for PE 2008-2013	117	101	Yes	No	Baseline, 1, 3, 6, 9, 12 months	AQOL SF6D FACT-C
Quyn 2016 ^44^	Australia	Prospective cohort	QoL, morbidity, survival	39	Palliative PE 1995-2015	30	Not known	No	No	Baseline, 1, 3, 6, 9, 12 months	AQOL SF-36
Cameron 2016 ^45^	Norway	Prospective cohort	Severity of symptoms	51	Palliative pelvic radio-therapy 2009-2015	12	Not known	No	No	Baseline, completion of radiotherapy, 6, 12 weeks	EORTC-C30 BPI
Pellino 2015 ^46^	Italy	Case-control	QoL	116	LRRC 2002-2011	45	40	Yes	Control group of patients with primary rectal cancer and R0 resection	Baseline, 12, 36 months	EORTC-C30
Li 2015 ^47^	China	Prospective cohort	Pain	31	LRRC 2009-2013	31	31	Yes	No	Baseline, 1 week, 1, 3, 6 months	VAS (pain)
Thaysen 2014 ^48^	Denmark	Case-control	QoL	180	PE 2001-2008	62	62	No	Compared to population norms and a group undergoing standard rectal cancer surgery.	Baseline, 3, 6, 12, 18, 24 months	EORTC-C30 & CR38 SF-36
Beaton 2014 ^49^	Australia	Cross-sectional	Morbidity, QoL	31	PE 1996-2007	17	17	No	Comparison of low, normal and high BMI	Cross-sectional	FACT-C
Pusceddu 2013 ^50^	Italy	Prospective cohort	Pain	12	LRRC with severe pain not responding to chemo-radiotherapy 2006-2010	12	12	Yes	No	Baseline, 1, 3, 6, 12, 22 months	VAS (pain)
Traa 2013 ^51^	Netherlands	Prospective cohort	QoL, sexual function	439	LARC and LRRC 2000-2010	67	67	Yes	Population norms vs LARC vs LRRC	Cross-sectional	EORTC-C30 & CR38
Holman 2013 ^52^	Netherlands	Cross-sectional	Flap-related complications, function following vaginal reconstruction, QoL	51	VRAM for LARC or LRRC 1994-2010	18	Not known	No	Patients with LARC and LRRC undergoing VRAM reconstruction vs patients not undergoing reconstruction.	Cross-sectional	EORTC-C30 & CR38
Brændengen 2011 ^53^	Norway	Cross-sectional	Morbidity, sexual function	207	Non-resectable LARC or LRRC undergoing pre-op radiotherapy or chemoradiotherapy 1996-2003	7	5	Yes	Patients receiving chemoradiotherapy vs those receiving radiotherapy	Cross-sectional	EORTC-C30 IIEF SVQ LENT SOMA St. Marks’s FI score
Haapamaki 2011 ^54^	Sweden	Cross-sectional	Physical function, QoL	19	Extralevator APER with gluteus maximus flap 2005-2007	1	1	No	No	Cross-sectional	EQ-5D EQ-VAS VAS
You 2011 ^33^	USA	Prospective cohort	Survival, QoL, Pain	105	LRRC 1997-2007	105	54	Yes	Curative treatment surgery vs non-curative surgery and non-surgical treatment	Baseline, 3, 6, 9, 12, 24, 36, 60, 96 months	FACT-C BPI
Austin 2010 ^55^	Australia	Case-control	QoL	44	PE 1996-2007	20	20	Yes	Patients undergoing PE vs patients with rectal cancer undergoing LAR or APER vs population norms	Cross-sectional	FACT-C SF-36
Zoucas 2010 ^56^	Sweden	Prospective cohort	Morbidity, survival, QoL	85	PE 2003-2008	20	Not known	No	No	4, 16 months	EORTC-C30
Palmer 2008 ^57^	Sweden	Case-control	QoL	142	LARC or LRRC 1991-2003	13	13	No	LARC and LRRC vs TME surgery alone and population norms	Cross-sectional	EORTC-C30 & CR38
Miner 2003 ^58^	USA	Prospective cohort	Morbidity, survival, QoL	105	LRRC 1997-1999	105	105	Yes	Palliative versus non-palliative treatment	Not specified	Not specified
Mannaerts 2002 ^59^	Netherlands	Prospective cohort	Functional outcome	121	LARC or LRRC 1994-1999	66	39	Yes	LARC vs LRRC	6 months pre-treatment, median 14 months post-treatment	Questionnaire devised for the study including questions from the anal incontinence scale and MSKCC Sphincter Function Scale
Esanaola 2002 ^60^	USA	Prospective cohort	Pain, QoL	45	LRRC 1999-2000	45	45	Yes	Non-operative palliation vs resection	Cross-sectional	FACT-C BPI
Camilleri-Brennan 2001 ^61^	UK	Cross-sectional	QoL	75	LRRC 1992-1997	13	13	No	LRRC vs patients with primary rectal cancer who did not develop recurrence	Cross-sectional	EORTC-C30 & CR38
Mannaerts 2001 ^62^	Netherlands	Retrospective cohort	Urological function	121	LARC or LRRC 1994-1999	66	39	Yes	LARC vs LRRC	Cross-sectional	Not specified
Guren 2001 ^63^	Norway	Case-control	QoL	37	Patients undergoing urinary diversion for LARC or LRRC since 1991	12	12	No	Patients undergoing urinary diversion vs patients who did not undergo urinary diversion vs population norms	Cross-sectional	EORTC-C30 & CR38 & BLM30 (6 items only)
Trotter 1996 ^64^	Australia	Randomised study	Disease progression, toxicity, QoL	73	LRRC or primary inoperable rectal cancer 1985-1991	64	64	No	Microwave therapy combined with external beam radiotherapy vs standard external beam radiotherapy	Weekly during treatment and then every 4 weeks	Spitzer
Scheithauer 1993 ^65^	Austria	Randomised study	Survival, QoL	36	Inoperable metastatic or recurrent colorectal cancer 1988-1989	Not known	Not known	No	Patients receiving chemotherapy vs best supportive care vs healthy volunteers	Baseline, every 2 months	FLIC
Wanebo 1987 ^66^	USA	Retrospective cohort	Morbidity, mortality, survival, QoL	28	LRRC	28	10	Yes	No	Cross-sectional	Not specified

*QoL* – quality of life, *PROM* – patient-reported outcome measure, *PE* - pelvic exenteration, *LRRC* – locally recurrent rectal cancer, *LARC* – locally advanced rectal cancer, *FACT-C* - Functional Assessment of Cancer Therapy – Colorectal Measure, SF-36 – 36-Item Short Form Survey, *TME* – total mesorectal excision, *LARS* – Low Anterior Resection Syndrome score, *EORTC*-C30 – European Organisation for Research and Treatment of Cancer Core Measure, *EORTC*-CR29/CR38 – European Organisation for Research and Treatment of Cancer Colorectal Module, *MSTS* – Musculoskeletal Tumour Society Score, *LEFS* – Lower Extremity Functional Scale, *SHIM* – Sexual Health Inventory for Men, *FSFI* – Female Sexual Function Index, *VRAM* - Vertical Rectus Abdominis Myocutaneous flap, *SBRT* – Stereotactic Body Radiotherapy, EQ-5D – EuroQoL measure of health-related quality of life, EQ-VAS – EuroQoL Visual Analogue Scale, *VNRS* – Verbal Numerical Rating Scale, *SF6D* – Short Form Six-Dimension, *AQOL* – Assessment of Quality of Life, *BPI* – Brief Pain Inventory, *R0* – Complete Surgical Resection, *VAS* – Visual Analogue Scale, *BMI* – Body Mass Index, *IIEF* – International Index of Erectile Function, *SVQ* – Sexual function – Vaginal changes Questionnaire, *LENT-SOMA* – Late Effects of Normal Tissue – Subjective , Objective, Management and Analytic, St. Mark’s FI Score – St. Mark’s Faecal Incontinence Score, *APER* – Abdominoperineal Excision of the Rectum, *LAR* – Low Anterior Resection, *MSKCC* – Memorial Sloan Kettering Cancer Center, EORTC-BLM30 – European Organisation for Research and Treatment of Cancer Muscle Invasive Bladder Cancer Measure, *FLIC* – Functional Living Index – Cancer.

### Risk of Bias

Risk of bias was high overall, with 32 (91.4%) studies highly or seriously biased (see supplementary Figures 1 and 2).

### Results of Individual Studies

#### Quality of Reporting of PROMs

**Fig. 3:**
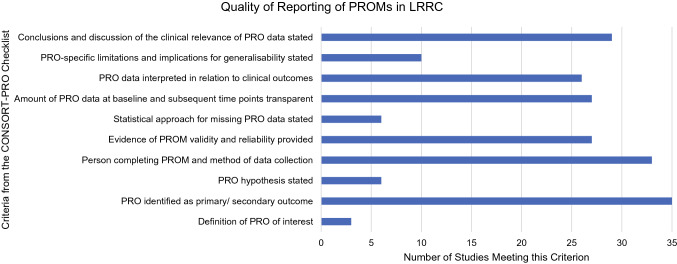
Quality of Reporting of PROMS in LRRC

The assessment of the studies identified against criteria informed by the CONSORT-PRO checklist are illustrated in Figure 3. None of the studies included in the review met all eleven criteria for the quality of reporting of PROMs, with an overall median of 5.8 (58.3%) criteria. The least reported criteria were defining the PROM of interest (*n *= 3, 8.6%), describing the statistical approach to missing PRO data (*n* = 6, 17.1%), and detailing a PRO hypothesis (*n *= 6, 17.1%). The most commonly met criterion was the identification of a PRO as a primary or secondary outcome (n = 35, 100.0%).

#### Characteristics of the PROMs Identified

Seventeen PROMs and two clinician-reported outcome measures (MSTS and Spitzer) were identified. The most commonly reported PROMs were the EORTC QLQ-C30 (*n *= 12, 32.3%),^34,36,45,46,48,51–53,56,57,61,63^ the SF-36 (*n *= 11, 31.4%),^7,35–38,40,41,43,44,48,55^ the FACT-C (*n *= 10, 28.6%)^7,33,35,37,40,41,43,49,55,60^ and the EORTC QLQ-CR29 (formerly CR38) (*n *= 8, 22.9%).^34,36,48,51,52,57,61,63^

Four of the PROMs identified were specific to patients with cancer (see Table 2), however, there were no disease-specific PROMs for patients with LRRC. The cancer-specific measures included the EORTC QLQ-C30 which is a measure of QoL in patients with cancer and the Functional Living Index – Cancer (FLIC) is a measure of functional state in adult patients with cancer. Two measures which are cancer-site specific were also identified; the EORTC-QLQ CR29 and FACT-C which are both measures of QoL in patients with primary colorectal cancer.

**Table 2:** Summary of cancer-specific measures identified

Measure	Patient-reported outcome	Target population	No of Items	Scales	No of languages/Dialogues	Total no of studies identified using this PROM	Studies identified using this PROM
European Organisation for Research and Treatment of Cancer Core Measure (EORTC QLQ-C30)	QoL	Patients with cancer	30	Functional scales: - Physical - Role - Cognitive - Emotional - Social Symptom scales: - Fatigue - Pain - Nausea and vomiting Global health status	117^67^	12	^34,36,45,46,48,51–53,56,57,61,63^
Functional Living Index – Cancer (FLIC)	Functional state	Patients with cancer	22	Psychological Physical Symptoms Family Social	15^68^	1	^65^
European Organisation for Research and Treatment of Cancer Colorectal Module (EORTC QLQ-CR29, formerly EORTC QLQ-CR38)	QoL	Patients with primary colorectal cancer	29	Urinary frequency Blood or mucus in stools Stool frequency Body image	66^69^	8	^34,36,48,51,52,57,61,63^
Functional Assessment of Cancer Therapy – Colorectal Measure (FACT-C)	QoL	Patients with primary colorectal cancer	36	Emotional Well-Being Social Well-Being Functional Well-Being Physical Well-Being Colorectal Cancer Subscale	40^70^	10	^7,33,35,37,40,41,43,49,55,60^

Seven PROMs which relate to forms of function or functional limitations were identified (Table 3), including bowel function, physical function, and sexual function. The Low Anterior Resection Syndrome (LARS) score is a measure to assess bowel dysfunction following low anterior resection for rectal cancer and the St. Mark’s Faecal Incontinence Score for adult patients with faecal incontinence. The Lower Extremity Functional Scale (LEFS) is a measure of lower extremity physical function designed for patients with lower extremity orthopaedic conditions. Four of the measures identified were measures of sexual function, including the Sexual Health Inventory for Men (SHIM) and the International Index of Erectile Function (IIEF) which are measures of erectile dysfunction developed for use in male patients with a history of erectile dysfunction and the Female Sexual Function Index (FSFI) measure of sexual function for female patients with a history of sexual arousal disorder and the Sexual function – Vaginal changes Questionnaire (SVQ) measure of sexual and vaginal problems developed for patients with a history of gynaecological cancer.

**Table 3:** Summary of measures related to function or functional limitations

Measure	Patient-reported outcome	Target population	No of items	Scales	No of languages/Dialogues	Total no of studies identified using this PROM	Studies identified using this PROM
Low Anterior Resection Syndrome (LARS) score	Low Anterior Resection Syndrome	Patients who have undergone low anterior resection for rectal cancer	5	N/A	24^71^	1	^34^
Lower Extremity Functional Scale (LEFS)	Lower extremity physical function	Patients with lower extremity orthopaedic conditions	20	N/A	14^68^	1	^36^
Sexual Health Inventory for Men (SHIM)	Erectile dysfunction	Male patients with erectile dysfunction	5	N/A	9^68^	1	^36^
International Index of Erectile Function (IIEF)	Erectile dysfunction	Male patients with erectile dysfunction	15	Erectile function Orgasmic function Sexual desire Intercourse satisfaction Overall satisfaction	88^68^	1	^53^
Female Sexual Function Index (FSFI)	Sexual function	Female patients with sexual arousal disorder	19	Desire Arousal Lubrication Orgasm Satisfaction pain	52^68^	1	^42^
Sexual function – Vaginal changes Questionnaire (SVQ)	Sexual and vaginal problems	Gynaecological cancer patients	20 core items (7 additional items for use in follow-up)	Intimacy Sexual interest Global sexual satisfaction Vaginal changes Sexual functioning	Not known	1	^53^
St. Mark’s Faecal Incontinence Score	Faecal incontinence	Adult patients with faecal incontinence	7	N/A	Not known	1	^53^

Six of the PROMs identified were generic measures (see Table 4), including three measures of QoL for use in adult patients; the 36-Item Short Form Survey (SF-36), EuroQoL (EQ-5D) and Assessment of Quality of Life (AQOL-4D), two measure of pain intensity; the Verbal Numerical Rating Scale (VNRS) and Visual Analogue Scale (VAS), and finally one measure of pain, the Brief Pain Inventory (BPI).

**Table 4:** Summary of generic measures identified

Measure	Patient-Reported Outcome	Target Population	No of Items	Scales	No of Languages/ Dialogues	Total no of studies identified using this PROM	Studies identified using this PROM
36-Item Short Form Survey (SF-36) including the Short Form Six-Dimension (SF6D)	QoL	Adult patients	36	Energy/ vitality Physical functioning Bodily pain General health perceptions Physical role functioning Emotional role functioning Social role functioning Mental health	2 available via RAND,^72^ 191 listed on ePROVIDE^68^	11	^34–38,40,41,43,44,48,55^
EuroQoL (EQ-5D) including the Visual Analogue Scale (EQ-VAS)	QoL	Adult patients	5	Mobility Self-care Usual activities Pain/ discomfort Anxiety/ depression	183^73^	2	^39,54^
Verbal Numerical Rating Scale (VNRS)	Pain Intensity	Adult patients	10-point scale	N/A	Not known	1	^42^
Visual Analogue Scale (VAS)	Pain Intensity	Adult patients	100mm line	N/A	Not known	3	^47,50,54^
Assessment of Quality of Life (AQOL-4D)	QoL	Adult patients	15	Illness Independent living Social relationships Physical senses Psychological wellbeing	7^74^	2	^43,44^
Brief Pain Inventory (BPI)	Pain	Adult patients	11	Pain intensity Pain interference	53^75^	3	^33,45,60^

The three remaining measures included (see Table 5), were not patient-reported but clinician reported. Those included the Late Effects of Normal Tissue – Subjective, Objective, Management, and Analytic (LENT-SOMA) scoring system for late effects of radiotherapy, including a subjective scale to be completed by patients with the remainder being completed by clinicians. The Spitzer is a clinician-reported measure of QoL for patients with cancer or other chronic diseases and the Musculoskeletal Tumour Society Score (MSTS) is a clinician-reported measure of physical function for patients with musculoskeletal neoplasms.

**Table 5:** Summary of other measures identified

Measure	Patient-reported Outcome	Target population	No of items	Scales	No of languages/Dialogues	Total no of studies identified using this PROM	Studies identified using this PROM
Late Effects of Normal Tissue – Subjective, Objective, Management, and Analytic (LENT-SOMA) scales	Late effects of radiotherapy	Adult patients who have received radiotherapy	5 (for subjective rectum scale)	Tenesmus Mucosal loss Sphincter control Stool frequency Pain	Not known	1	^53^
Spitzer *designed to be used as a clinician-reported outcome measure	QoL	Patients with cancer or other chronic diseases	5	Activity Daily life Health perceptions Social support Behaviour	5^68^	1	^64^
Musculoskeletal Tumour Society Score (MSTS) *designed to be used as a clinician-reported outcome measure	Physical function	Patients with musculoskeletal neoplasms	6	Pain Function Emotional acceptance Criteria specific to the lower extremity: - Use of supports - Walking - Gait Criteria specific to the upper extremity: - Hand positioning - Manual dexterity - Lifting ability	Not known	1	^36^

### PROM Psychometric Properties

The psychometric properties were only assessed for PROMs and not the LENT-SOMA or the clinician-reported outcome measures, Spitzer and MSTS.

#### Content Validity

None of the PROMs identified were developed specifically for patients with LRRC (Tables 2, 3, 4 and 5) and no studies were identified in which the psychometric properties of these PROMs were evaluated in patients with LRRC.

#### Internal Structure and Remaining Measurement Properties

Content validity is the most important measurement property of a PROM and therefore full review is not advised if a PROM does not meet criteria for content validity.

## Discussion

There has been an expansion in PROMs reporting in LRRC, with several papers (*n *= 21, 63.6%) published in the last decade. However, despite this increase, these studies are methodologically limited due to the use of non-validated measures used to assess PROs in this cohort of patients. This systematic review did not identify a disease-specific PROM available for use in LRRC and none of the PROMs identified met the COSMIN criteria for content validity in the context of LRRC. The most used PROMS in LRRC were the FACT-C (*n *= 10, 28.6%), SF-36 (*n* = 11, 31.4%) EORTC QLQ-C30 (*n *= 12, 34.3%) and CR29 (*n *= 8, 22.9%), none of which have demonstrated content validity specifically for patients with LRRC.

Overall, the findings build on the existing evidence^16–19^ of variable methodological quality of reporting of PROMs within small sample sizes and mixed disease cohorts. This review focuses specifically on the methodological quality of PRO reporting using criteria informed by the CONSORT-PRO checklist; common weaknesses were identified in several domains, including defining the PRO of interest, describing the statistical approach to missing data and stating PRO-specific limitations and implications for generalisability. These results were comparable to those reported in Efficace et al.’s pooled analysis of randomised cancer trials utilising CONSORT-PRO,^76^ though methods of PRO data collection had higher levels of reporting in this current review. Ultimately, the key limitation identified is the lack of input from patients with LRRC in the PROMs currently being used, with none demonstrating content validity for use in this context. Content validity is the most important measurement property of a PROM; for PROMs to give meaningful results in LRRC, it is essential that they are relevant to patients with LRRC and present a comprehensive assessment of the construct of interest. Without addressing the lack of an appropriate PROM for use in patients with LRRC, the impact of addressing issues such as heterogeneity in the groups of patients included, the comparator groups used, and the timing of PROM assessment, is likely to be limited.

Harji et al. reported the development of the Locally Recurrent Rectal Cancer – Quality of Life (LRRC-QoL) conceptual framework through undertaking a systematic review and qualitative focus groups to identify the HrQoL issues relevant to patients with LRRC.^18,77^ The themes identified were symptoms, sexual function, psychological impact, role and social functioning, future perspective and healthcare service utilisation and delivery. Nineteen (54.3%) of the studies identified in this review have been published since this work,^35–51^ using a median of two PROMS, with the EORTC QLQ-CR29 and FACT-C most used. The EORTC QLQ-CR29 and FACT-C have also both demonstrated robust psychometric properties, including content validity, in patients with primary colorectal cancer.^78,79^ When compared with the LRRC-QoL conceptual framework,^77^ the EORTC QLQ-CR29 covers 50% of the LRRC-specific domains, including symptoms, sexual function, and psychological impact. It does not however cover the domains of role functioning, or future perspective. The FACT-C covers 66.6% of the LRRC-specific domains identified in the LRRC-QoL conceptual framework including symptoms, psychological impact, role functioning, and future perspective, it does not cover sexual function. Neither the EORTC QLQ-CR29 or FACT-C cover issues relating to healthcare services, self-efficacy and body image, future plans, disease re-recurrence, gynaecological or locomotor symptoms. The evidence identified reporting outcomes utilising these PROMs should not be completely disregarded, as the EORTC QLQ-CR29 and FACT-C capture a proportion of the issues relevant to patients with LRRC. However, it should be interpreted with caution, as they are unlikely to capture the full scope and complexity of the range of issues patients with LRRC experience.^18,77^

A number of PROMs which measure issues relevant to patients with LRRC were identified in this review; urinary and sexual function were evaluated using specific questionnaires for this purpose by two studies,^36,53^ however, other questionnaires, such as the EORTC QLQ-CR29, also contain items concerning sexual and urinary function. No specific PROMs concerning stoma-related quality of life were used in the studies identified, despite being relevant to patients with LRRC.^77^ However, PROMs such as the EORTC QLQ-CR29 and FACT-C contain items specifically for patients with stomas. The increasing number of PROMs currently being used in LRRC reflects the lack of an existing disease-specific measure which adequately reports all the PROs relevant to this cohort of patients. The trend to include several PROMs is likely to reflect the greater understanding of the wider issues which affect patients with LRRC. However, the measures identified in this review are not valid for use in patients with LRRC and therefore this is not a psychometrically robust approach to addressing the lack of a LRRC disease-specific measure. Additionally, this approach potentially increases the burden of participation for patients, without sufficient methodological justification.

There are limitations related to the evidence included in this review, notably, most of the studies identified have a high risk of bias (*n *= 32, 91.4%) and their findings should generally be interpreted with caution. They also present a predominately Western perspective of PROs in LRRC and demonstrate a lack of multi-centre, international reporting of PROs in LRRC. Furthermore, 13 (37.1%) of the studies identified were conducted within a single centre, reporting cohorts of patients which may potentially overlap. It was not possible to assess the availability and quality of translated PROMs in this review, however, to further the success of initiatives such as the PelvEx collaborative in advancing international outcome reporting in this cohort of patients^80^ and integrating PRO data, it is essential that PROMs undergo a rigorous process of cross-cultural adaption.

There are several approaches which could be employed to address the lack of PROMs with content validity for patients with LRRC. It is possible to demonstrate the content validity of existing PROMS specifically for LRRC, however, given the narrow breadth of relevant HrQoL issues captured by existing measures, this approach will require significant revision to make these measures applicable to LRRC.^77^ Employing a modular approach to PROM assessment to LRRC is an alternative approach, provided both the core cancer and site-specific measures are appropriately revised and validated for use in LRRC. Development of a new disease-specific PROMs for use in patients with LRRC, to capture concerns that are specific to patients with LRRC which can be used to more accurately monitor the impact of particular treatments on PROs such as HrQoL is likely to be the most realistic and valid approach.^81^ The development of the LRRC-QoL PROM will build on the development of the LRRC-QoL conceptual framework.^77^ The LRRC-QoL is the first disease-specific PROM developed for use in patients with LRRC^82^ and has been designed to be used in combination with EORTC QLQ-C30, in a modular fashion, which would allow comparison across patient groups. Recruitment to a study to externally validate the LRRC-QoL for use internationally is currently underway (ISRCTN13692671) and includes a robust cross-cultural adaptation process to produce versions of the LRRC-QoL for use in several countries.

## Conclusion

This systematic review highlights key methodological issues in the current state of reporting of PROs in LRRC, finding that none of the PROMs currently being used in LRRC are able to provide meaningful results within this context. Future studies in this disease area should focus on utilising PROMs that have undergone a robust development process with the inclusion of patients with LRRC, to ensure high quality, accurate results which are relevant to this patient group. The development of a disease-specific PROM for patients with LRRC or undertaking content validity studies of existing PROMs are approaches which could be employed to enable this, in addition to undertaking cross-cultural adaptation to enable international reporting of outcomes. Greater emphasis should also be placed on the way in which PROMs data are reported and analysed, particularly in defining the PRO of interest and in handling missing PROM data, to ensure that results are reliable.

## Supplementary Information

Below is the link to the electronic supplementary material.

Supplementary file1 (DOCX 339 KB)

Supplementary file2 (DOCX 53 KB)
