# Supplementary material for: Systematic Review of Patient-Reported Outcome Measures in Locally Recurrent Rectal Cancer
Source: Ann Surg Oncol. 2023 Apr 18;30(7):3969–86. doi: 10.1245/s10434-023-13388-5 (PMC10250265; doi:10.1245/s10434-023-13388-5)
Supplement: Supplementary file 1 — Supplementary file1 (DOCX 339 KB) [file 10434_2023_13388_MOESM1_ESM.docx]

**Supplemental Materials**

Figure 1: ROBINS-I risk of bias for observational studies


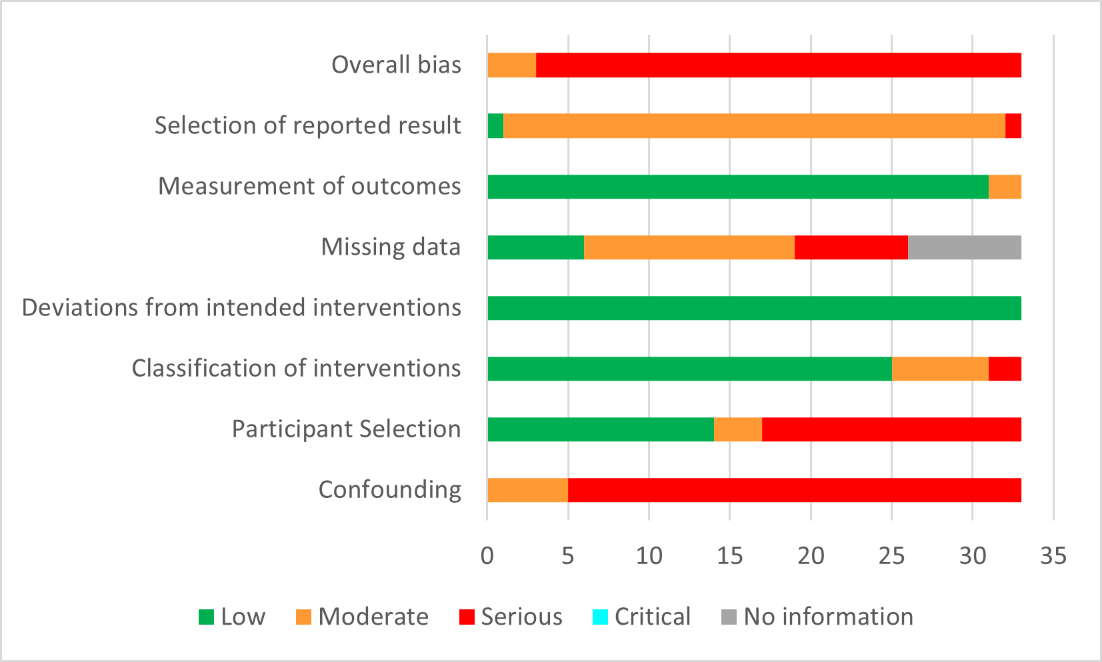


Figure 2: RoB 2 risk of bias for randomised studies


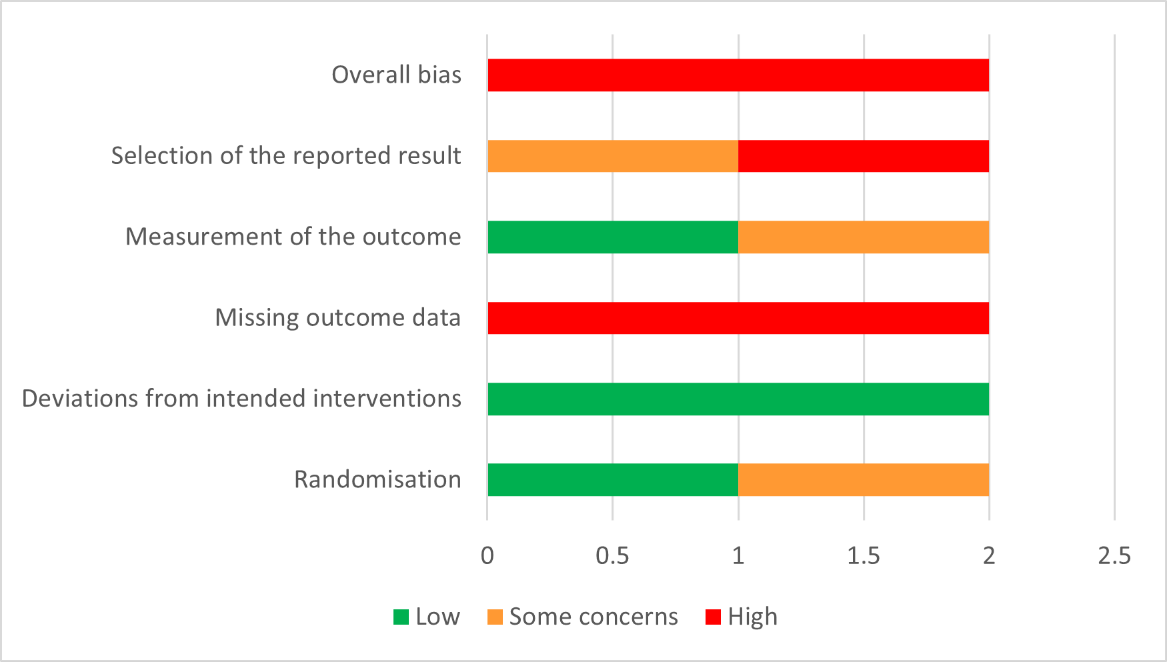


**Systematic Review Search Strategy**

(locally AND recurrent AND (rectal neoplasm [MeSH Terms] OR cancer of the rectum OR rectal cancer OR rectal tumour))

AND

(((patient-reported AND outcome*) OR (patient-reported AND outcome* AND measure) OR (PROM*))

OR

((quality of life [MeSH Terms] OR (quality AND life) OR quality of life) OR (health-related quality of life OR ((health [MeSH Terms] OR health) AND related AND quality of life))

OR

(symptom AND prevention and control [Subheading])

OR

(questionnaires [MeSH Terms] OR questionnaires)).

**Evaluation of PROMs Against COSMIN Criteria for Content Validity in the Context of the Patient Group for which they were Developed**

The psychometric properties were only assessed for PROMs and not the LENT-SOMA or the clinician-reported outcome measures, Spitzer and MSTS.

**Content validity**

None of the PROMs identified were developed specifically for patients with LRRC and no studies were identified in which the psychometric properties of these PROMs were evaluated in patients with LRRC. A pragmatic decision was therefore undertaken to assess content validity in relation to the specific group in which the PROM had been developed to gain an understanding of the overall quality of the PROMs being used in LRRC. Content validity was deemed adequate for five PROMs, when assessed in the context of the specific subset of patients for which they had been developed. All other PROMs did not meet criteria for content validity.

The PROMs which did meet the criteria for content validity included the FACT-C, which was developed as a measure of QoL in patients with primary colorectal cancer^1^, the EORTC QLQ-C30, which was developed as a measure of QoL in patients with cancer and was initially developed in a cohort of patients with lung cancer^2^, the EORTC QLQ-CR29, which was developed as a measure of QoL in patients with primary colorectal cancer^3^, the EQ-5D-5L was developed as a generic measure of QoL through focus groups including healthy participants and those with chronic disease^4^, finally the SF-36, which was developed as a generic measure of QoL in patients with chronic conditions and was initially developed in a cohort of patients with diabetes, hypertension, heart disease and/or depression^5,6^. All five PROMs demonstrated moderate to high quality evidence for the three aspects of content validity assessed: relevance, comprehensiveness, and comprehensibility. However, it is worth noting that reporting of assessment for comprehensiveness could generally be improved by describing the methods undertaken more explicitly.

In terms of the PROMs which did not meet the COSMIN criteria for content validity, the Sexual Health Inventory for Men (SHIM) is an abridged version of the International Index of Erectile Function (IIEF). The IIEF development included interviews with patients with erectile dysfunction and their partners, the IIEF did not meet the COSMIN criteria for content validity due to not being described in sufficient detail^7^. The Sexual function – Vaginal changes Questionnaire (SVQ) development included interviews with patients with gynaecological cancer, it was given an inconsistent rating for relevance, an insufficient rating for comprehensiveness and a sufficient rating for comprehensibility^8^. The Female Sexual Function Index (FSFI) was developed for patients with female sexual arousal disorder and its development included interviews with this group of patients in addition to female volunteers from the general population, the measure was given an inconsistent rating for relevance and insufficient ratings for both comprehensiveness and comprehensibility^9^.

The Lower Extremity Functional Scale (LEFS) involved patients with a history of lower-extremity musculoskeletal dysfunction (defined as any condition of the joints, muscles, or other soft tissues) in the process of item development, however there was no evidence that its relevance, comprehensiveness, and comprehensibility have been established in the population of interest and it was therefore rated as insufficient for content validity^10^. The St. Mark’s Faecal Incontinence Score did not meet the criteria for relevance, comprehensibility or comprehensiveness due to not involving patients with faecal incontinence, who were the population of interest, in the development process^11^. The Low Anterior Resection Syndrome (LARS) score was developed to assess bowel dysfunction in patients who had undergone low anterior resection for rectal cancer, the LARS score demonstrated good evidence for comprehensibility and comprehensiveness. However, it was rated inconsistent in relation to the criteria for relevance, due to not involving patients who had undergone low anterior resection in the initial process of item generation^12^. The Functional Living Index – Cancer (FLIC) development involved interviews with patients, though the characteristics of these patients were not described, this was followed by review of the items by a panel which included one male and one female patient and two patient spouses^13^. Ultimately it was rated as inconsistent for relevance and insufficient for both comprehensiveness and comprehensibility.

In terms of the generic measures identified, the psychometric properties of the Assessment of Quality of Life (AQoL-4D) were assessed as this is the version referenced^14^, this measure was developed through interviews with a range of medical conditions. The AQoL-4D did not meet the COSMIN criteria for content validity particularly due to a lack of evidence demonstrating comprehensiveness or comprehensibility, however newer versions have since been developed and validated^15^. It was not possible to evaluate the content validity of the Visual Analogue Scale (VAS) or Verbal Numerical Rating Scale (VNRS) given the nature of these single-item measures of pain intensity, in addition to the VAS has been in use for a century^16^. It was also not possible to evaluate the BPI fully due to being unable to retrieve the full text for the PROM development study^17^, ratings were therefore determined from the development of the first version of the BPI, namely the Wisconsin Brief Pain Questionnaire^18^ and did not meet criteria for content validity due to lack of sufficient evidence.

**Internal structure and remaining measurement properties**

Content validity is the most important measurement property of a PROM and therefore full review is not advised if a PROM does not meet criteria for content validity. A summary of the findings for the internal structure and remaining measurement properties of the five PROMs which were deemed to meet the criteria for content validity can be found in Table 1.

Table 1: Quality of the evidence for the measurement properties of the PROMs – FACT-C, EORTC QLQ-C30, EORTC QLQ-CR29, EQ-5D and SF-36

|  | **FACT-C** | | **EORTC QLQ-C30** | | **EORTC QLQ-CR29** | | **EQ-5D-5L** | | | **SF-36** | |
| --- | --- | --- | --- | --- | --- | --- | --- | --- | --- | --- | --- |
|  | **Overall rating** | **Quality of Evidence** | **Overall rating** | **Quality of Evidence** | **Overall rating** | **Quality of Evidence** | **Overall rating** | **Quality of Evidence** | **Overall rating** | | **Quality of Evidence** |
|  | **+ / - / ?** | **High, moderate, low, very low.** | **+ / - / ?** | **High, moderate, low, very low.** | **+ / - / ?** | **High, moderate, low, very low.** | **+ / - / ?** | **High, moderate, low, very low.** | **+ / - / ?** | | **High, moderate, low, very low.** |
| **Content validity** | + | Moderate | + | High | + | High | + | High | + | | High |
| Relevance | + | High | + | High | + | High | + | High | + | | High |
| Comprehensiveness | + | Moderate | + | High | + | High | + | High | + | | High |
| Comprehensibility | + | Moderate | + | High | + | High | + | High | + | | High |
| **Structural validity** | + | High | + | High | + | High | + | High | + | | High |
| **Internal consistency** | + | High | + | High | + | High | N/A | N/A | + | | High |
| **Reliability** | + | High | ? | Moderate | + | High | ? | Moderate | ? | | Moderate |
| **Construct validity** | + | High | + | Moderate | + | High | + | High | + | | Moderate |
| **Responsiveness** | ? | Low | ? | Moderate | ? | Moderate | ? | Low | + | | High |

**References**

**1.** Ward W, Hahn E, Mo F, Hernandez L, Tulsky D, Cella D. Reliability and validity of the Functional Assessment of Cancer Therapy-Colorectal (FACT-C) quality of life instrument. *Quality of Life Research.* 1999;8(3):181-195.

**2.** Aaronson NK, Ahmedzai S, Bergman B, et al. The European Organization for Research and Treatment of Cancer QLQ-C30: A Quality-of-Life Instrument for Use in International Clinical Trials in Oncology. *JNCI: Journal of the National Cancer Institute.* 1993;85(5):365-376.

**3.** Gujral S, Conroy T, Fleissner C, et al. Assessing quality of life in patients with colorectal cancer: An update of the EORTC quality of life questionnaire. *European Journal of Cancer.* 2007/07/01/ 2007;43(10):1564-1573.

**4.** Herdman M, Gudex C, Lloyd A, et al. Development and preliminary testing of the new five-level version of EQ-5D (EQ-5D-5L). *Qual Life Res.* 2011;20(10):1727-1736.

**5.** Tarlov AR, Ware JE, Jr., Greenfield S, Nelson EC, Perrin E, Zubkoff M. The Medical Outcomes Study: An Application of Methods for Monitoring the Results of Medical Care. *JAMA.* 1989;262(7):925-930.

**6.** Ware JE, Jr., Sherbourne CD. The MOS 36-item short-form health survey (SF-36). I. Conceptual framework and item selection. *Med Care.* Jun 1992;30(6):473-483.

**7.** Rosen RC, Riley A, Wagner G, Osterloh IH, Kirkpatrick J, Mishra A. The international index of erectile function (IIEF): a multidimensional scale for assessment of erectile dysfunction. *Urology.* Jun 1997;49(6):822-830.

**8.** Jensen PT, Klee MC, Thranov I, Groenvold M. Validation of a questionnaire for self-assessment of sexual function and vaginal changes after gynaecological cancer. *Psycho-Oncology.* 2004/08/01 2004;13(8):577-592.

**9.** Rosen C, Brown J, Heiman S, et al. The Female Sexual Function Index (FSFI): A Multidimensional Self-Report Instrument for the Assessment of Female Sexual Function. *Journal of Sex & Marital Therapy.* 2000/04/01 2000;26(2):191-208.

**10.** Binkley JM, Stratford PW, Lott SA, Riddle DL. The Lower Extremity Functional Scale (LEFS): Scale Development, Measurement Properties, and Clinical Application. *Physical Therapy.* 1999;79(4):371-383.

**11.** Vaizey CJ, Carapeti E, Cahill JA, Kamm MA. Prospective comparison of faecal incontinence grading systems. *Gut.* 1999;44(1):77-80.

**12.** Emmertsen KJ, Laurberg S. Low Anterior Resection Syndrome Score: Development and Validation of a Symptom-Based Scoring System for Bowel Dysfunction After Low Anterior Resection for Rectal Cancer. *Annals of Surgery.* 2012;255(5).

**13.** Schipper H, Clinch J, McMurray A, Levitt M. Measuring the quality of life of cancer patients: the Functional Living Index-Cancer: development and validation. *Journal of Clinical Oncology.* 1984/05/01 1984;2(5):472-483.

**14.** Hawthorne G, Richardson J, Osborne R. The Assessment of Quality of Life (AQoL) instrument: a psychometric measure of Health-Related Quality of Life. *Quality of Life Research.* 1999/05/01 1999;8(3):209-224.

**15.** Richardson JRJ, Peacock SJ, Hawthorne G, Iezzi A, Elsworth G, Day NA. Construction of the descriptive system for the assessment of quality of life AQoL-6D utility instrument. *Health and Quality of Life Outcomes.* 2012/04/17 2012;10(1):38.

**16.** Couper MP, Tourangeau R, Conrad FG, Singer E. Evaluating the Effectiveness of Visual Analog Scales: A Web Experiment. *Social Science Computer Review.* 2006/05/01 2006;24(2):227-245.

**17.** Cleeland CS, Ryan KM. Pain assessment: global use of the Brief Pain Inventory. *Ann Acad Med Singap.* Mar 1994;23(2):129-138.

**18.** Daut RL, Cleeland CS, Flanery RC. Development of the Wisconsin Brief Pain Questionnaire to assess pain in cancer and other diseases. *Pain.* 1983/10/01/ 1983;17(2):197-210.
