# Supplementary material for: Systematic Review of Patient-Reported Outcome Measures in Locally Recurrent Rectal Cancer
Source: Ann Surg Oncol. 2023 Apr 18;30(7):3969–86. doi: 10.1245/s10434-023-13388-5 (PMC10250265; doi:10.1245/s10434-023-13388-5)
Supplement: Supplementary file 2 — Supplementary file2 (DOCX 53 KB) [file 10434_2023_13388_MOESM2_ESM.docx]

**Identification of studies via databases and registers**

Records identified from*:

PubMed (n = 1128)

Embase (n = 272)

CINAHL (n = 75)

Records removed *before screening*:

**Duplicate records removed (n = 147)**

**Records removed for other reasons (n = 5)**

**Identification**

Records excluded**

Studies not including patients with LRRC (n= 664)

Studies in patients with LRRC not including PROMs (n = 122)

Reviews/letters/guidelines (n = 365)

Case reports (n = 47)

Video abstract (n = 2)

Surveys/Delphi of healthcare practitioners (n = 22)

Protocols (n = 24)

Conference abstract (n = 21)

Records screened

(n = 1323)

Reports not retrieved

(n = 8)

Reports sought for retrieval

(n = 56)

**Screening**

Reports excluded:

Only includes patients with LARC (n = 1)

Does not include PROMs (n = 16)

Reports assessed for eligibility

(n = 48)

Manual searching:

Papers identified (n = 4)

Studies included in review

(n = 35)

**Included**

*Consider, if feasible to do so, reporting the number of records identified from each database or register searched (rather than the total number across all databases/registers).

**If automation tools were used, indicate how many records were excluded by a human and how many were excluded by automation tools.

*From:*  Page MJ, McKenzie JE, Bossuyt PM, Boutron I, Hoffmann TC, Mulrow CD, et al. The PRISMA 2020 statement: an updated guideline for reporting systematic reviews. BMJ 2021;372:n71. doi: 10.1136/bmj.n71

For more information, visit: <http://www.prisma-statement.org/>
